# Carnitine and COVID-19 Susceptibility and Severity: A Mendelian Randomization Study

**DOI:** 10.3389/fnut.2021.780205

**Published:** 2021-11-25

**Authors:** Chunyu Li, Ruwei Ou, Qianqian Wei, Huifang Shang

**Affiliations:** Department of Neurology, Laboratory of Neurodegenerative Disorders, National Clinical Research Center for Geriatrics, West China Hospital, Sichuan University, Chengdu, China

**Keywords:** carnitine, COVID-19, protective, Mendelian randomization (MR), causation

## Abstract

**Background:** Carnitine, a potential substitute or supplementation for dexamethasone, might protect against COVID-19 based on its molecular functions. However, the correlation between carnitine and COVID-19 has not been explored yet, and whether there exists causation is unknown.

**Methods:** A two-sample Mendelian randomization (MR) analysis was conducted to explore the causal relationship between carnitine level and COVID-19. Significant single nucleotide polymorphisms from genome-wide association study on carnitine (*N* = 7,824) were utilized as exposure instruments, and summary statistics of the susceptibility (*N* = 1,467,264), severity (*N* = 714,592) and hospitalization (*N* = 1,887,658) of COVID-19 were utilized as the outcome. The causal relationship was evaluated by multiplicative random effects inverse variance weighted (IVW) method, and further verified by another three MR methods including MR Egger, weighted median, and weighted mode, as well as extensive sensitivity analyses.

**Results:** Genetically determined one standard deviation increase in carnitine amount was associated with lower susceptibility (OR: 0.38, 95% CI: 0.19–0.74, *P*: 4.77E−03) of COVID-19. Carnitine amount was also associated with lower severity and hospitalization of COVID-19 using another three MR methods, though the association was not significant using the IVW method but showed the same direction of effect. The results were robust under all sensitivity analyses.

**Conclusions:** A genetic predisposition to high carnitine levels might reduce the susceptibility and severity of COVID-19. These results provide better understandings on the role of carnitine in the COVID-19 pathogenesis, and facilitate novel therapeutic targets for COVID-19 in future clinical trials.

## Introduction

Coronavirus disease 2019 (COVID-19), caused by the severe acute respiratory syndrome coronavirus 2 (SARS-CoV-2), has spread across the world and led to substantial morbidity and mortality (1). Global efforts have been invested in treatment options concerning the pandemic, but no effective therapy has been found so far. Although a number of drugs have been put in clinical trials, evidence from a large living systematic review and network meta-analysis suggested that only glucocorticoids, such as dexamethasone, probably reduce mortality and mechanical ventilation in patients with severe COVID-19 (2). Coincidentally, L-carnitine, a potentially promising substitute for dexamethasone, has been found to mimic some of the biological activities of glucocorticoids, especially immunomodulation, while with less side effect (3). L-Carnitine was shown to have antiviral and anti-inflammatory effects in HCV and HIV infections (4, 5). Meanwhile, L-carnitine is an essential nutrient with a major role in cellular energy production (6), while abnormal lipid metabolism is a common symptom of COIVD-19 (7). Furthermore, earlier investigations have suggested that L-carnitine might be a supportive and therapeutic option to prevent the harm caused by COVID-19 based on its molecular functions (8, 9). However, the correlation between carnitine and COVID-19 has not been explored yet, and whether there exists causation is still unknown.

Here, to evaluate the correlation between carnitine level in the body and risk of COVID-19, we employed the two-sample Mendelian randomization (MR) approach to explore the causal role of carnitine on COVID-19 (10, 11). As a result, we found that higher carnitine level was causally associated with decreased susceptibility and severity of COVID-19.

## Methods

### Datasets

We obtained summary statistics for carnitine from a previous genome-wide association study (GWAS) on human metabolic traits (12). This study analyzed the genetic influences on human blood metabolites using the GWAS method in 7,824 European-ancestry participants. The study design like the collection of samples, quality control procedures and imputation methods have been described in the original publication. Single nucleotide polymorphisms (SNP) that passed the generally accepted genome-wide significance threshold (*P* < 5E−08) for carnitine were chosen as instrument variants. None of the significant SNPs for carnitine was suggestively associated with body mass index (BMI) searched in GWAS Catalog (13) (*P* < 1E−05). Then we clumped instrument variables based on 1,000 Genomes Project linkage disequilibrium (LD) structure, and kept index SNPs (*R*^2^ < 0.001 with any other associated SNP within 10 Mb) with the lowest *P*-value. The final summary information for each instrument was shown in Supplementary Tables 1–3.

Summary statistics of COVID-19 susceptibility and severity for the selected instrument variables were obtained from the COVID-19 Host Genetics Initiative (14) (https://www.covid19hg.org/, Release 5). GWAS on COVID-19 susceptibility involved 42,557 patients with COVID-19 and 1,424,707 population controls, while GWAS on COVID-19 severity involved 5,582 very severe respiratory confirmed patients with COVID-19 and 709,010 population controls. Meanwhile, we also analyzed GWAS on COVID-19 hospitalization involving 9,986 hospitalized patients with COVID-19 and 1,877,672 population controls, since hospitalized patients with COVID-19 were mostly with severe symptoms. Harmonization was undertaken to rule out strand mismatches and ensure alignment of SNP effect sizes. The final summary information was shown in Supplementary Tables 1–3.

The current study only utilized publicly available summarized results from published genome-wide association studies. No individual-level data were involved.

### Mendelian Randomization Analysis

We hypothesized that carnitine level as a protective factor could causally decrease the risk of COVID-19, and the following assumptions were satisfied: the genetic variants used as instrumental variables are associated with carnitine level; the genetic variants are not associated with any confounders; the genetic variants are associated with COVID-19 through carnitine level (namely horizontal pleiotropy should not be present).

To comprehensively evaluate the causative effect of carnitine on COVID-19, we performed the two-sample MR analysis using the random effects inverse variance weighted (IVW) method, which is most widely used in MR studies and could provide robust causal estimates under the absence of directional pleiotropy. Meanwhile, we verified the results using another three MR methods, namely Mendelian randomization Egger regression, weighted median and weighted mode. To evaluate potential violations of the model assumptions in the MR analysis, we further conducted comprehensive sensitivity analyses. Cochran's Q test was computed to check heterogeneity across the individual causal effects. Mendelian Randomization Pleiotropy RESidual Sum and Outlier (MR-PRESSO) analysis was conducted to detect outlier instrument (15). MR-Egger regression was performed to evaluate the directional pleiotropy of instruments (16). To evaluate the strength of each instrument variable, we computed the F-statistic of each SNP as described by a previous study (17). Leave-one-out analysis was conducted with the inverse variance weighted method to assess the influence of individual variants on the observed association. And reverse causal inference was conducted to explore whether COVID-19 susceptibility and severity have a causal impact on carnitine level with Steiger analysis (18). The statistical power calculated at http://cnsgenomics.com/shiny/mRnd/ is sufficient (1.00) assuming the true causal OR of carnitine on COVID-19 is 0.8, given the involved sample size and the significance level α as 0.05 (19). The main statistical analyses were conducted using the R package TwoSampleMR 0.5.5 (20).

## Results

**Figure 1:**
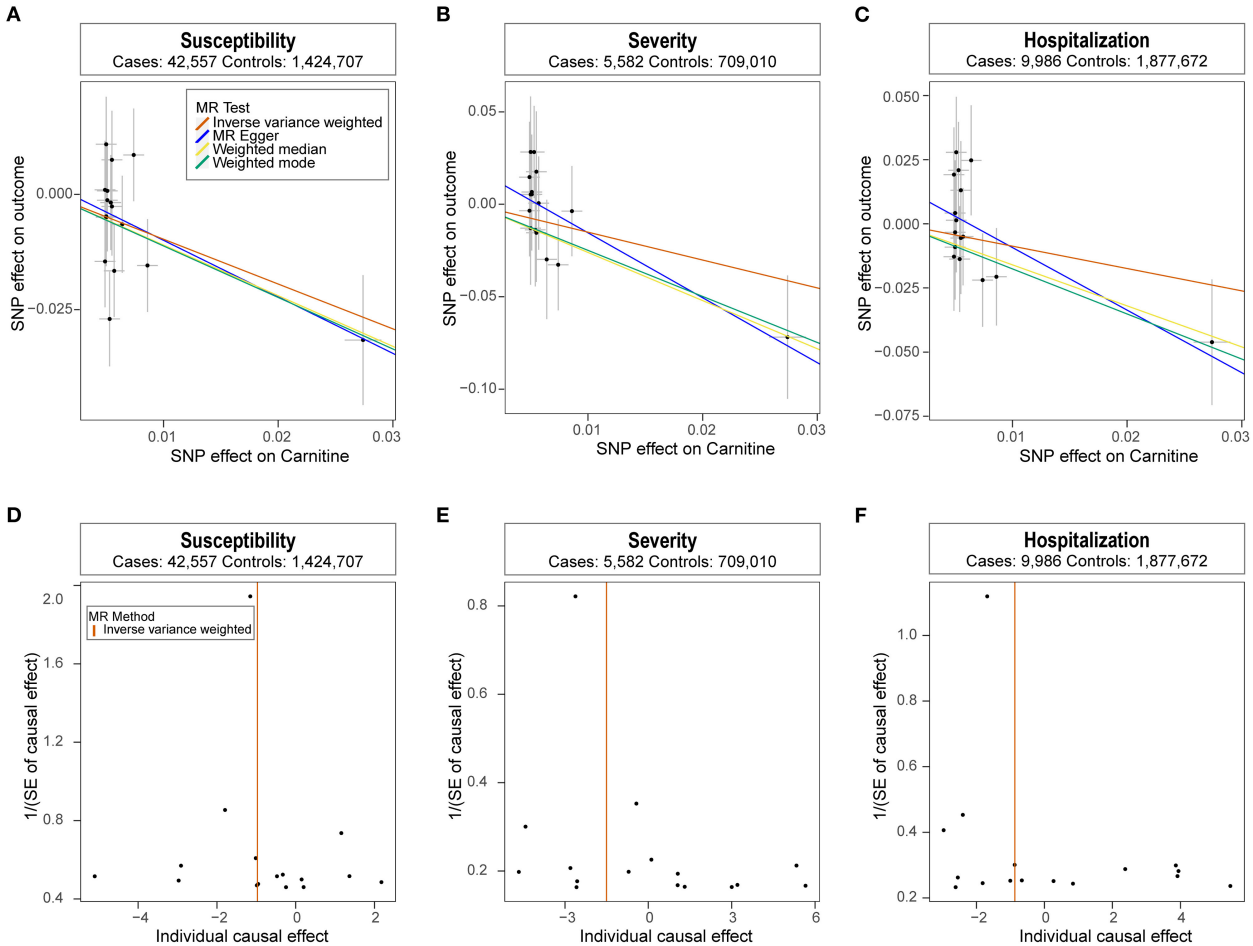
Mendelian randomization analysis results. **(A-C)** Scatter plot of single nucleotide polymorphism (SNP) potential effects on carnitine level vs. COVID-19. The 95% CI for the effect size on COVID-19 is shown as vertical lines, while the 95% CI for the effect size on carnitine level is shown as horizontal lines. The slope of fitted lines represents the estimated Mendelian randomization (MR) effect per method. **(D-F)** Forest plot showing results from the Mendelian randomization analysis to evaluate potential causal associations between carnitine and COVID-19. Estimates are per 1 standard deviation (SD) increase in the trait.

We analyzed the role of carnitine on COVID-19 using the two-sample MR approach. Results showed that each one standard deviation (1-SD) increase in carnitine amount was associated with a lower risk of COVID-19 (OR: 0.38, 95% CI: 0.19~0.74, P: 4.77E−03), and the results were verified using the weighed median and weighted mode methods (Figure 1A). Meanwhile, higher carnitine amount was associated with lower severity and hospitalization of COVID-19 using three MR methods, though such association was not significant using the IVW method (Figures 1B,C). The funnel plot displays symmetric pattern of effect size variation around the point estimate (Figures 1D–F).

Next, we performed extensive sensitivity analyses to validate the causal association between carnitine and COVID-19. No heterogeneity of effects was detected using Cochran's Q test (Table 1). The F statistics of all the instrument variables were above 10 (ranging from 29 to 293), indicating the absence of weakness in the selected instruments. The intercept of MR-Egger is not significantly deviated from zero, suggesting no apparent horizontal pleiotropy (Table 1). Directionality examination by Steiger analysis did not suggest a violation of the causality either. The MR-PRESSO analysis detected no potential instrumental outlier at the nominal significance level of 0.05. The leave-one-out results suggest that the causal effect was not driven by single instrumental variable (Supplementary Figures 1, 2).

**Table 1:** Heterogeneity and horizontal pleiotropy analyses results.

**Outcome**	**Heterogeneity**		**Horizontal pleiotropy**			
	**IVW Q**	**IVW Q df**	**IVW *P* value**	**Egger intercept**	**SE**	***P***
COVID-19 susceptibility	14.49	15	0.49	0.0023	0.01	0.65
COVID-19 severity	7.80	15	0.93	0.020	0.01	0.14
COVID-19 hospitalization	11.27	15	0.73	0.015	0.01	0.11

*IVW, inverse variance weighted; Q, Cochran's Q test estimate; df, Cochran's Q test degrees of freedom; SE, standard error*.

## Discussion

Recent studies have proposed the potential role of carnitine as therapy options for COVID-19, but whether there is any correlation between them has not been explored yet. Meanwhile, unmeasured confounding factors in clinical studies can potentially bias the association evidence, as is a common criticism inherent to observational studies. Here, based on results from comprehensive two-sample MR analyses, we demonstrated that higher carnitine level was causally associated with decreased susceptibility and severity of COVID-19.

Carnitine occurs in two forms known as D-carnitine and L-carnitine, and only L-carnitine is biologically active in the body (21). L-carnitine is an essential carrier for long-chain fatty acids from the cytosol through the inner mitochondrial membrane into the matrix, where β-oxidation takes place (22). Although no studies have investigated the correlation between carnitine and COVID-19, previous research on carnitine might provide some insights from molecular aspects of why L-carnitine might protect against COVID-19. L-carnitine plays an important role in fatty acid metabolism and could act as an adjuvant agent in the improvement of dyslipidemia (23). L-carnitine was previously found to increase high-density lipoprotein and lower triglyceride, total cholesterol and low-density cholesterol (23), while dyslipidemia has been shown to be associated with the risk and severity of COVID-19 repeatedly (7, 24). In addition, as an effective antioxidant, L-carnitine was involved in modulating the mechanisms of the immune system and the nervous system (8), and could inhibit the expression of inflammatory factors (25, 26). Antioxidants supplementation has been recommended in therapeutic strategies against COVID-19 (27), since antioxidant therapy could improve oxygenation rates, glutathione levels and strengthen the immune response (28). Meanwhile, anti-inflammatory drugs were suggested to potentially inhibit a key enzyme in the replication and transcription of SARS-CoV-2 (29), and anti-inflammatory agents have also been proposed as potential therapies for COVID-19 due to their prevention of cardiovascular events. Furthermore, L-carnitine plays a critical role in energy production, as it transports long-chain fatty acids into the mitochondria so they can be oxidized to produce energy (30). More energy for the immune system means more immune cells can be produced to protect against infection from virus. Lastly, current evidence showed that severely ill patients with COVID-19 tend to have a high concentration of pro-inflammatory cytokines, such as IL-6, compared to those who are moderately ill, and the high level of cytokines also indicates a poor prognosis in COVID-19 (31). L-carnitine could suppress the production of pro-inflammatory cytokines by preventing the hyperosmolarity-induced oxidative stress (32), and thus might help prevent patients with COVID-19 from cytokine storm. Taken together, so many biological functions make L-carnitine a potential therapeutic option to protect against COVID-19. Unfortunately, no clinical or epidemiological studies have investigated the correlation between them. Here, using the MR approach, we clarified the protective role of carnitine on COVID-19 susceptibility and severity from a genetic perspective. Future clinical or functional studies could put importance to this, and further explore the role of carnitine in protecting against COVID-19.

Based on the Mendelian randomization results obtained from large-scale GWAS summary data, we demonstrated that carnitine might have a protective role on COVID-19, and carnitine might be a therapy which is worth further exploration in clinical trials. These results help better understand the role of carnitine on COVID-19, and will facilitate therapeutic drugs in future clinical trials.

## Strengths and Weaknesses

The strength of our work is the Mendelian randomization method which could avoid confounding factors. And comprehensive sensitivity analyses guaranteed the reliability of the association. Meanwhile, current results have very important clinical implications. Up till now, global efforts were still invested in finding effective drugs for COVID-19. Our findings provided some guidance and new direction for future clinical trials. There were also some limitations to the current study. Though we identified a protective role of carnitine against COVID-19, the biological mechanism of this protection was still unclear. Further clinical and functional studies were necessary to clarify the effect of carnitine on COVID-19.

## Data Availability Statement

Summary statistics of carnitine could be found in http://mips.helmholtz-muenchen.de/proj/GWAS/gwas/index.php (ID: M15500). Summary statistics of COVID-19 could be downloaded from the COVID-19 Host Genetics Initiative (https://www.covid19hg.org/, release 5). The datasets used and/or analyzed during the current study are available from the corresponding author on reasonable request. Code or algorithm used to generate results in this study are available from the corresponding authors on reasonable request.

## Author Contributions

CL and HS conceived the study. CL performed the statistical analyses and prepared the drafted manuscript. RO, QW, and HS contributed to writing and editing of the manuscript. All authors reviewed and approved the final manuscript.

## Funding

This study was supported by the funding of the National Natural Science Foundation of China (81871000 and 81901294), the China Postdoctoral Science Foundation (2019M653424), the 1.3.5 Project for Disciplines of Excellence, West China Hospital, Sichuan University (ZYJC18038), the National Clinical Research Center for Geriatrics, West China Hospital, Sichuan University (Grant No. Z20192006), and the Science Foundation of Chengdu Science and Technology Bureau (2019-YF05-00307-SN).

## Conflict of Interest

The authors declare that the research was conducted in the absence of any commercial or financial relationships that could be construed as a potential conflict of interest.

## Publisher's Note

All claims expressed in this article are solely those of the authors and do not necessarily represent those of their affiliated organizations, or those of the publisher, the editors and the reviewers. Any product that may be evaluated in this article, or claim that may be made by its manufacturer, is not guaranteed or endorsed by the publisher.

## Abbreviations

COVID-19: coronavirus disease 2019
SARS-CoV-2: severe acute respiratory syndrome coronavirus 2
MR: Mendelian randomization
GWAS: genome-wide association study
SNP: single nucleotide polymorphism
LD: linkage disequilibrium
SD: standard deviation
IVW: inverse variance weighted.
