# Supplementary material for: Carnitine and COVID-19 Susceptibility and Severity: A Mendelian Randomization Study
Source: Front Nutr. 2021 Nov 25;8:780205. doi: 10.3389/fnut.2021.780205 (PMC8656944; doi:10.3389/fnut.2021.780205)
Supplement: Supplementary file 1 [file Data_Sheet_1.PDF]

Supplementary Figure 1. Forest plot of the association between significant SNPs of carnitine and COVID-19, together with pooled estimates. (**A**) COVID-19 susceptibility. (**B**) COVID-19 severity. (**C**) COVID-19 hospitalization.

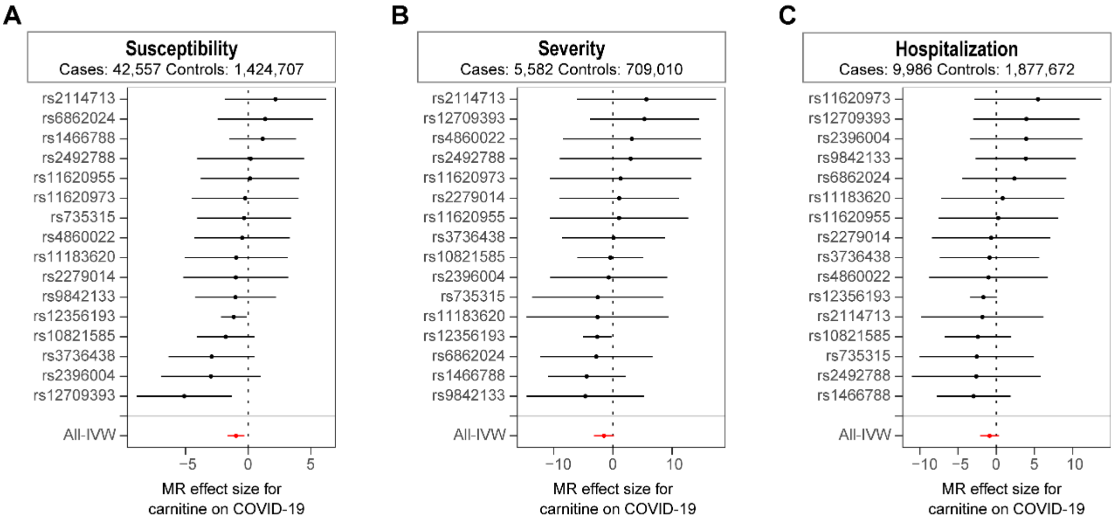

Supplementary Figure 2. Forest plot of the results of the leave-one-out sensitivity analysis, where each SNP in the instrument was iteratively removed from the instrument variables. (**A**) COVID-19 susceptibility. (**B**) COVID-19 severity. (**C**) COVID-19 hospitalization.

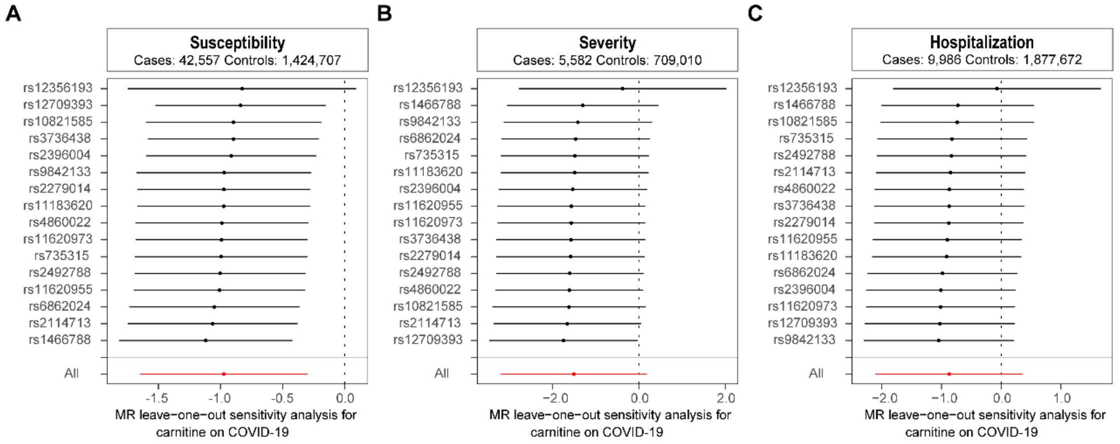

Supplementary Table 1. Summary statistics for instrumental variables of carnitine for COVID-19 susceptibility

SNP	A1	A2	carnitine			COVID-19 susceptibility		
			**beta**	**P value**	**SE**	**beta**	**P value**	**SE**
rs10821585	A	G	-8.60E-03	1.28E-21	9.00E-04	1.54E-02	0.126	0.010
rs11183620	A	G	5.00E-03	3.00E-08	9.00E-04	-4.76E-03	0.650	0.010
rs11620955	A	G	-5.10E-03	1.63E-08	9.00E-04	-7.65E-04	0.940	0.010
rs11620973	A	G	-5.10E-03	2.35E-08	9.00E-04	1.26E-03	0.910	0.011
rs12356193	A	G	2.74E-02	3.69E-63	0.0016	-3.16E-02	0.025	0.014
rs12709393	T	G	5.30E-03	6.41E-09	9.00E-04	-2.70E-02	0.009	0.010
rs1466788	A	G	-7.40E-03	3.05E-16	9.00E-04	-8.56E-03	0.394	0.010
rs2114713	T	G	-5.00E-03	2.67E-08	9.00E-04	-1.09E-02	0.291	0.010
rs2279014	T	C	-5.00E-03	4.86E-08	9.00E-04	4.93E-03	0.643	0.011
rs2396004	A	G	4.90E-03	4.32E-08	9.00E-04	-1.46E-02	0.142	0.010
rs2492788	T	C	4.90E-03	4.58E-08	9.00E-04	9.82E-04	0.927	0.011
rs3736438	T	G	-5.70E-03	3.19E-10	9.00E-04	1.66E-02	0.097	0.010
rs4860022	T	C	-5.50E-03	9.60E-10	9.00E-04	2.62E-03	0.806	0.011
rs6862024	A	G	-5.50E-03	8.99E-10	9.00E-04	-7.50E-03	0.482	0.011
rs735315	T	C	5.40E-03	1.87E-09	9.00E-04	-1.77E-03	0.864	0.010
rs9842133	T	C	6.40E-03	4.20E-12	9.00E-04	-6.49E-03	0.538	0.011

SNP, single nucleotide polymorphism; A1, effect allele; A2, other allele; beta, SNP effect size; SE, standard error of the SNP effect size. All the selected instruments together explain about 11.2% phenotypic variation of carnitine at the observed scale.

Supplementary Table 2. Summary statistics for instrumental variables of carnitine for COVID-19 severity

SNP	A1	A2	carnitine			COVID-19 severity		
			beta	P value	SE	beta	P value	SE
rs10821585	A	G	-8.60E-03	1.28E-21	9.00E-04	3.70E-03	0.879	0.024
rs11183620	A	G	5.00E-03	3.00E-08	9.00E-04	-1.29E-02	0.673	0.031
rs11620955	A	G	-5.10E-03	1.63E-08	9.00E-04	-5.38E-03	0.859	0.030
rs11620973	A	G	-5.10E-03	2.35E-08	9.00E-04	-6.68E-03	0.830	0.031
rs12356193	A	G	2.74E-02	3.69E-63	0.0016	-7.19E-02	0.031	0.033
rs12709393	T	G	5.30E-03	6.41E-09	9.00E-04	2.83E-02	0.257	0.025
rs1466788	A	G	-7.40E-03	3.05E-16	9.00E-04	3.27E-02	0.184	0.025
rs2114713	T	G	-5.00E-03	2.67E-08	9.00E-04	-2.83E-02	0.345	0.030
rs2279014	T	C	-5.00E-03	4.86E-08	9.00E-04	-5.30E-03	0.837	0.026
rs2396004	A	G	4.90E-03	4.32E-08	9.00E-04	-3.47E-03	0.888	0.025
rs2492788	T	C	4.90E-03	4.58E-08	9.00E-04	1.47E-02	0.623	0.030
rs3736438	T	G	-5.70E-03	3.19E-10	9.00E-04	-6.26E-04	0.980	0.025
rs4860022	T	C	-5.50E-03	9.60E-10	9.00E-04	-1.76E-02	0.589	0.033
rs6862024	A	G	-5.50E-03	8.99E-10	9.00E-04	1.54E-02	0.562	0.027
rs735315	T	C	5.40E-03	1.87E-09	9.00E-04	-1.38E-02	0.651	0.031
rs9842133	T	C	6.40E-03	4.20E-12	9.00E-04	-2.98E-02	0.356	0.032

SNP, single nucleotide polymorphism; A1, effect allele; A2, other allele; beta, SNP effect size; SE, standard error of the SNP effect size. All the selected instruments together explain about 11.2% phenotypic variation of carnitine at the observed scale.

Supplementary Table 3. Summary statistics for instrumental variables of carnitine for COVID-19 hospitalization

SNP	A1	A2	carnitine			COVID-19 hospitalization		
			beta	P value	SE	beta	P value	SE
rs10821585	A	G	-8.60E-03	1.28E-21	9.00E-04	2.06E-02	0.277	0.019
rs11183620	A	G	5.00E-03	3.00E-08	9.00E-04	4.19E-03	0.838	0.021
rs11620955	A	G	-5.10E-03	1.63E-08	9.00E-04	-1.37E-03	0.946	0.020
rs11620973	A	G	-5.10E-03	2.35E-08	9.00E-04	-2.79E-02	0.197	0.022
rs12356193	A	G	2.74E-02	3.69E-63	0.0016	-4.61E-02	0.059	0.024
rs12709393	T	G	5.30E-03	6.41E-09	9.00E-04	2.09E-02	0.267	0.019
rs1466788	A	G	-7.40E-03	3.05E-16	9.00E-04	2.19E-02	0.228	0.018
rs2114713	T	G	-5.00E-03	2.67E-08	9.00E-04	9.09E-03	0.656	0.020
rs2279014	T	C	-5.00E-03	4.86E-08	9.00E-04	3.33E-03	0.866	0.020
rs2396004	A	G	4.90E-03	4.32E-08	9.00E-04	1.92E-02	0.297	0.018
rs2492788	T	C	4.90E-03	4.58E-08	9.00E-04	-1.28E-02	0.544	0.021
rs3736438	T	G	-5.70E-03	3.19E-10	9.00E-04	4.99E-03	0.792	0.019
rs4860022	T	C	-5.50E-03	9.60E-10	9.00E-04	5.53E-03	0.800	0.022
rs6862024	A	G	-5.50E-03	8.99E-10	9.00E-04	-1.31E-02	0.494	0.019
rs735315	T	C	5.40E-03	1.87E-09	9.00E-04	-1.38E-02	0.504	0.021
rs9842133	T	C	6.40E-03	4.20E-12	9.00E-04	2.47E-02	0.248	0.021

SNP, single nucleotide polymorphism; A1, effect allele; A2, other allele; beta, SNP effect size; SE, standard error of the SNP effect size. All the selected instruments together explain about 11.2% phenotypic variation of carnitine at the observed scale.
